# Against Over-reliance on PRISMA Guidelines for Meta-analytical Studies

**DOI:** 10.5041/RMMJ.10518

**Published:** 2024-01-19

**Authors:** Jaime A. Teixeira da Silva, Timothy Daly

**Affiliations:** 1 Independent Researcher, Kagawa-ken, Japan; 2 Bioethics Program, FLACSO Argentina, Buenos Aires, Argentina; 3 Science Norms Democracy, Sorbonne Université, Paris, France

**Keywords:** Diversity, editorial responsibility, equity and inclusion, ethics, meta-analysis, systematic review

## Abstract

The Preferred Reporting Items for Systematic Reviews and Meta-Analyses (PRISMA) guidelines were elaborated to allow authors of such papers to identify quality articles for inclusion in their scholarly work. However, we have identified several issues that point to an over-reliance on the PRISMA guidelines. Firstly, we question the rigor of implementation by authors and the rigor of verification by peer reviewers and editors, and whether they have screened papers to ensure adherence to the PRISMA guidelines. Secondly, we have identified cases where the PRISMA criteria led to as much as 99.97% of the published literature being ignored, suggesting that valid publications meeting these criteria might be at risk of being ignored. Thirdly, we have noted that exclusion is not only a quantitative problem—it is also a qualitative one, since the screening procedure groups all non-conforming literature into one basket. Fourthly, we have noted that seven copies of the PRISMA guidelines exist. This being the case, which one should be cited? To replace over-reliance on PRISMA screening, we encourage authors, peer reviewers, and editors to publish systematic reviews and meta-analyses that respect the dual criteria of scientific plausibility and diversity of included papers.

## INTRODUCTION

Meta-analytic studies in the form of systematic reviews and meta-analyses (SR+MAs) of existing studies are a powerful tool to generate cumulative knowledge and improve the robustness of conclu-sions aimed at answering specific questions, pro-vided they do not succumb to the cherry-picking of studies, i.e. publication bias.^1^ For instance, with regard to the coronavirus disease pandemic, there were instances of individual low-quality studies, with meta-analytic studies based upon them being used to push an anti-scientific agenda.^2^

The Preferred Reporting Items for Systematic Reviews and Meta-Analyses (PRISMA) guidelines were initially written in 2009^3^ and followed by a revised version in 2021.^4^ The guidelines were aimed at offering a rigorous methodology for authors of SR+MAs to meticulously select studies and litera-ture providing substantial evidence for inclusion.^4^ Designed for the medical sciences, the PRISMA guidelines are supposed to allow compliant SR+MAs to deliver only the best and most robust available medical evidence to readers, policy-makers, and healthcare providers. The PRISMA 2020 statement comprises a checklist and a flowchart.^5^ The checklist comprises 27 main items and several sub-items, intended to guide users as to what steps should be taken to make their SR+MAs PRISMA-compliant.^6^ The almost “standardized” flowchart is provided for SR+MA authors to modify and present in their pa-pers. In theory and on paper, the PRISMA checklist and accompanying flowchart seem incorruptible, fair, inclusive, unbiased, and comprehensive. How-ever, we have identified four potential issues that contribute to what we consider to be an over-reliance on the guidelines.

## ISSUE #1: RIGOR OF IMPLEMENTATION

The first issue we touch upon is the rigor of PRISMA guidelines implementation by authors, as well as the rigor of verification of its implementation by review-ers and journal editors. To address this point, we turn to the critique of Arab-Zozani and Hassani-pour^7^ regarding a study by Hasanpour Dehkordi et al.^8^ Hasanpour Dehkordi et al. claimed to respect PRISMA guidelines but did not actually “adhere to its components.”^7^ For example, it was ambiguous as to whether the article was a systematic review or a meta-analysis; the number of articles included was not mentioned; there was no mention of database search strategies; and the order for PRISMA report-ing was not respected.^7^ In other words, authors may claim that their study is PRISMA-compliant, merely to satisfy the journal’s editorial requests or to im-prove the publishability of their study, without actu-ally rigorously striving for utmost quality in their meta-analytical study.

## ISSUE #2: EXCLUSION CRITERIA

Second, we argue that there seems to be a discrep-ancy in academic research between, on the one hand, a researcher’s strong reliance on the PRISMA guidelines with very stringent exclusionary criteria and, on the other hand, publishers and journals that advocate for diversity, equity, and inclusion (DEI) policies. This raises an important question: Is the prioritization of robust evidence in the PRISMA selection procedure compatible with DEI policies?

We believe that over-dependence on the PRISMA criteria can lead to excessive literary exclusion. Re-ferring only to the number of papers excluded from SR+MAs, we assessed five SR+MAs published in 2022 and indexed in PubMed. We noted that these reviews disregarded a substantial proportion of the available literature, as much as 97%–99% (Table 1). Hence, based on our assessment, these SR+MAs were lacking in “knowledge inclusivity.”

**Table 1:** Examples of Five Systematic Reviews and Meta-analyses with High Exclusion Rates that Claimed PRISMA-2020 Compliance.

DOI	Original Dataset	Excluded Papers	Final Included Papers (%)
10.1016/j.jclinepi.2022.06.021	30,592	30,565	27 (0.09%)
10.1093/heapro/daac078	2321	2261	60 (2.59%)
10.1093/rheumatology/keac500	4364	4331	33 (0.76%)
10.1136/bmj-2022-072003	7229	7154	75 (1.04%)
10.1371/journal.pone.0270494	1574	1549	25 (1.59%)

## ISSUE #3: HOMOGENIZATION OF EXCLUDED STUDIES

Third, PRISMA screening treats all non-conforming literature the same. Excluded studies are put togeth-er in one basket without distinction—regardless of whether they are irrelevant, fake, unsound, non-robust, or any other category—and portrays them all negatively. Just as it is inappropriate for a field scientist to disregard their responsibility to truthfully consider inconvenient data,^9^ authors of SR+MAs should similarly avoid publishing meta-analytical conclusions that fail to accurately portray reality or encompass the entirety of the published literature.

Claiming PRISMA compliance can serve as a convenient pretext to include and assess only a few dozen studies in an SR+MA, as opposed to having to try and accommodate several hundred or thousand. We believe that as many thematically relevant studies as possible should be included. However, the weaknesses of those studies that might initially be excluded due to PRISMA implementation should be emphasized; if word or page limits exist, these can easily be presented as a supplementary file.

However, in this context, authors are not the sole agents responsible for ensuring that the published scientific record of SR+MAs is representative of the published literature. We consider peer reviewers and editors to also be complicit in a phenomenon of “PRISMA signaling”—akin to virtue signaling—leading to a showcasing of PRISMA compliance, while at the same time not fully adhering to the PRISMA guidelines. Expert peer reviewers of SR+MA manuscripts should endeavor to remind authors of important papers that may have been disregarded due to a standardized PRISMA approach. In turn, editors can remind peer reviewers to ensure appro-priate PRISMA use, facilitating a shared responsi-bility for appropriate use of the PRISMA guidelines (Table 2).

**Table 2:** Pro-active Suggestions for Authors, Peer Reviewers, and Editors to Ensure Appropriate Use of PRISMA Guidelines in Systematic Reviews and Meta-analyses.

Systematic Reviews and Meta-Analyses		
Authors	Peer Reviewers	Editor Considerations
Avoid standardized use of PRISMA guidelines	Screen included and excluded sets carefully	Mention PRISMA over-reliance in the journal instructions for authors
Avoid PRISMA signaling	Detect PRISMA-signaling	Adopt a flexible editorial policy for SR+MAs
Include raw datasets of included and excluded studies	Mention missing papers	Publish raw datasets of included and excluded studies

## ISSUE #4: MULTIPLE COPIES OF GUIDELINES

The fourth issue that we have noted admittedly dovetails with other issues beyond the scope of this particular commentary. Nevertheless, it represents a problem that will be encountered by anyone wanting to cite the PRISMA guidelines. A recent search for PRISMA on PubMed—a popular public database dedicated almost exclusively to the biomedical sci-ences—revealed only one copy of the original 2009 guidelines; on the other hand, seven copies of the 2020 official guidelines were noted, five that were textually identical and two translations—one in Spanish and another in Portuguese (Table 3). Admit-tedly, a search on Google Scholar or other estab-lished proprietary databases, such as Web of Science or Scopus, might have revealed different findings. Scientists may not be aware of the existence of the PRISMA website, or they may seek to cite the PRISMA guidelines that appear in a peer-reviewed paper rather than citing its website. When academics wish to cite the PRISMA guidelines,^4^ which of these copies of the guidelines should they select? We opted for the *British Medical Journal* copy simply because it was the first one listed in PubMed, although we note that Sohrabi et al.^10^ opted to cite the statement published in their own journal, *International Journal of Surgery*, while Parums^11^ opted for the *PLOS Medicine* copy. A wider debate on cloned guidelines and robust systematic analyses of their impact on citation patterns is merited.

**Table 3:** Copies of the PRISMA-2020 Guidelines Found in PubMed.

DOI	PubMed URL
10.1136/bmj.n71	https://pubmed.ncbi.nlm.nih.gov/33782057/
10.1016/j.jclinepi.2021.03.001	https://pubmed.ncbi.nlm.nih.gov/33789819/
10.1016/j.rec.2021.07.010	https://pubmed.ncbi.nlm.nih.gov/34446261/
10.1016/j.ijsu.2021.105906	https://pubmed.ncbi.nlm.nih.gov/33789826/
10.1371/journal.pmed.1003583	https://pubmed.ncbi.nlm.nih.gov/33780438/
10.1186/s13643-021-01626-4	https://pubmed.ncbi.nlm.nih.gov/33781348/
10.26633/RPSP.2022.112	https://pubmed.ncbi.nlm.nih.gov/36601438/

## CONCLUSION

We conclude that attempting to rigorously filter the literature using PRISMA guidelines to include only the most evidence-based and data-robust studies in SR+MAs may have unintended and undesirable re-sults. These might include “literature discrimina-tion” (i.e. non-inclusivity) and a practice akin to data cherry-picking.^12^ Such an approach, in our view, contradicts the DEI policies currently existent in academic publishing. Instead, we propose that authors, peer reviewers, and editors should publish SR+MAs that employ both scientific plausibility and plurality as criteria for selecting papers. This ap-proach aligns with a more democratic vision for con-tributing to scientific knowledge, aimed at avoiding monolithic thinking.^13^

The problems highlighted in this commentary are not meant to criticize the existence of the PRISMA guidelines; rather, they address the over-reliance on these guidelines by authors of meta-analytical studies or SR+MAs. It also addresses the potential lack of robust screening procedures by peer reviewers and editors to ensure that relevant literature has not been unfairly excluded.

## Abbreviations

PRISMA: Preferred Reporting Items for Systematic Reviews and Meta-Analyses
SR+MAs: systematic reviews and meta-analyses
